# An Investigation on Energy Harvesting Behavior of an Array Piezoelectric Coupled Disc Damper

**DOI:** 10.3390/mi13081244

**Published:** 2022-08-02

**Authors:** Xiangdong Xie, Xunnan Huang, Junjie Wang, Zijing Wang, Bowen Zhou, Jiankun Zhang

**Affiliations:** School of Urban Construction, Yangtze University, Jingzhou 434000, China; 15027174591@163.com (X.H.); wangjunjie4316@163.com (J.W.); 15071562521@163.com (Z.W.); z2443353863@163.com (B.Z.); zjk9709@163.com (J.Z.)

**Keywords:** piezoelectric damper, excitation frequency, energy harvesting behavior, peak-to-peak voltage, piezoelectric coupled disc

## Abstract

In order to make full use of the vibration energy in the process of attenuating vibration, an array piezoelectric coupled disc damper is developed, which works by converting part vibration energy into electrical energy. The piezoelectric damper is made of a pair of piezoelectric coupled discs built in a case cylinder. Its energy harvesting behavior is studied by a series of forced-vibration experiments and simulations. The influences of some factors, such as the excitation frequency, substrate thickness, the size of the piezoelectric patch, the paste form of the piezoelectric patch and the load resistance, on the energy harvesting behavior of the damper are analyzed and concluded. The experimental results show that the maximum peak-to-peak voltage and average power from one piezoelectric patch with an inner diameter of 35 mm, an outer diameter of 80 mm, and a thickness of 1 mm can reach up to 163 V and 161 mW, respectively. This research provides a practical piezoelectric damper attenuating harmful vibration by converting them into useful electric power, and the corresponding theoretical models are derived to predict its electrical output.

## 1. Introduction

Due to the action of energy dissipation and vibration absorption, dampers are widely used in mechanical manufacture, civil engineering, automotive industry, and other fields. The traditional dampers, usually including liquid dampers, friction dampers, and regenerative dampers, have been researched and applied widely in different fields for many years [1,2,3,4].

Liquid damper, for its low initial capital, easy installation, almost no maintenance, and easy to match the tuned frequency, has been studied and used in various devices, such as bridges [5], buildings [6,7,8], wind turbines [9] and so on. Wang et al. [10] proposed a damping system by embedding tuned liquid multiple column damper into reinforced concrete shear wall systems to improve the energy dissipation capabilities of traditional structural component. Rozas et al. [11] presented a bidirectional tuned liquid column damper to reduce the seismic response of buildings. Zhang et al. [12] evaluated the performance of a full-scale tuned liquid damper in mitigating lateral tower vibrations of multi-megawatt wind turbines. In addition, Sarkar and Chakraborty [13], Zhang and Høeg [14] and Chen et al. [15] developed different liquid dampers, which all displayed a good effect in controlling the vibration. However, the thing with liquid damper is, its dissipated damping is often affected by the environment temperature. In addition, in order to enhance the robustness, multiple viscous liquid dampers are needed at the same time, which causes the complicated structure and the higher cost. Therefore, researchers devoted their interest to friction dampers with a steady property and simple structure. Latour et al. [16] proposed a removable friction damper for steel beam-to-column joints to improve the moment resisting ability of the steel frames. Ghorbani and Rofooei [17] developed a novel double slip loads friction damper to control the seismic response of structures, which can effectively reduce the seismic response of the structures compared with the traditional single slip load friction damper. Furthermore, Pardo-Varela and Llera [18], and Chen et al. [19,20] also provided their respective friction damper to reduce the seismic response of the structures in different aspects. All dampers aforesaid can achieve a high level of vibration dissipation. However, they cannot transfer the vibration energy into electric power for recycling applications. In view of this, a series of regenerative dampers are developed, which have a good efficiency in dissipating vibration while achieving an efficient energy harvesting by electromagnetic technology. Li et al. [21] modeled two types of rotational electromagnetic regenerative dampers used for vehicle suspension. The research results show that both dampers can make vehicles more comfortable in the driving process. Meanwhile, the dampers can generate considerable electric power outputs for the reuse of vehicles. Li et al. [22], Gao et al. [23], Sung and Choi [24], respectively, developed their electromagnetic dampers and applied them to the suspension of the vehicle. In addition, Li et al. [25] and Jung et al. [26] fabricated their electromagnetic dampers suitable to the cable structures, the research results reveal that the dampers had a good ability to control vibration and harvest energy simultaneously. There are also many scholars, such as Li and Zhu [27], Hu et al. [28], Luo et al. [29], and Kopylov et al. [30] proposed their own dampers by different ways, which can effectively transfer the vibration into electricity through electromagnetic technology.

Although the aforementioned regenerative dampers can convert some of the vibration energy of devices into electric energy in the attenuation process, the conversion mechanism of these dampers is electromagnetic transaction which has a lower energy conversion density compared with the piezoelectric transaction [31,32]. Meanwhile, the piezoelectric materials can be widely coupled with different structures to achieve piezoelectric transaction [33,34,35,36,37]. Yoon et al. [38] proposed a PVDF–silicone rubber damper using to attenuate the mutual collision of the offshore structures and studied the deformation and energy harvesting behavior of the sandwiched and coiled sheet geometries of the device. The experiment results show that the output power density of the coiled-type damper can reach up to 0.982 mW/cm^3^. Khan and Ali [39] presented a vibration isolator composed of a piezoelectric disc embedded in the silicone rubber. This isolator can convert the environmental vibration into electrical energy, and it can obtain an optimum power of 2.12 mW at a matching load of 340 kΩ and frequency (resonant) of 56 Hz. However, the devices above cannot efficiently harvest the vibration energy and just generate a small amount of electric energy.

In view of this, in this research, a practical piezoelectric damper attenuating harmful vibration by converting them into useful electric power, and the corresponding theoretical models are derived to predict its electrical output. The novel damper is made of a pair of linear arrays of piezoelectric coupled discs built-in a case cylinder, and the theoretical models are based on a series of experiments and simulations. According to the test results, the average power (power density) can reach up to 161.03 mW (39.6 mW/cm^3^) from a piezoelectric patch. Correspondingly, the total output power of the piezoelectric damper consisting of 20 unimorph PCDs can reach up to 3.22 W, which can serve for many electric appliances such as sensors, actuators, signal transmitters.

## 2. Development of the Piezoelectric Damper

**Figure 1:**
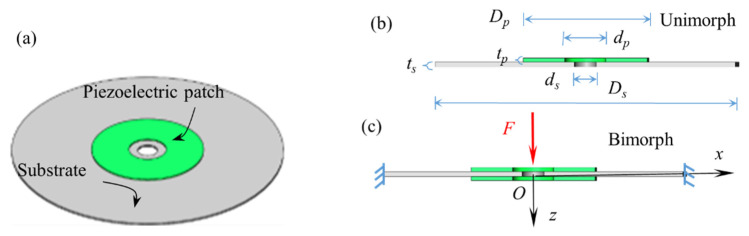
Set-up of a piezoelectric coupled disc (PCD). (**a**) 3D view of PCD; (**b**) normal section view of unimorph PCD; (**c**) normal section view of bimorph PCD.

**Figure 2:**
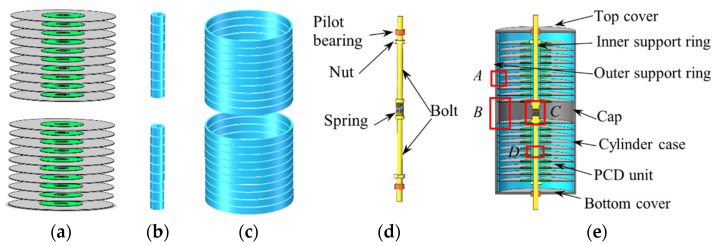
Set-up of a piezoelectric damper. (**a**) PCD array; (**b**) inner support ring array; (**c**) outer support ring array; (**d**) load transfer unit; (**e**) section view of piezoelectric damper.

**Figure 3:**
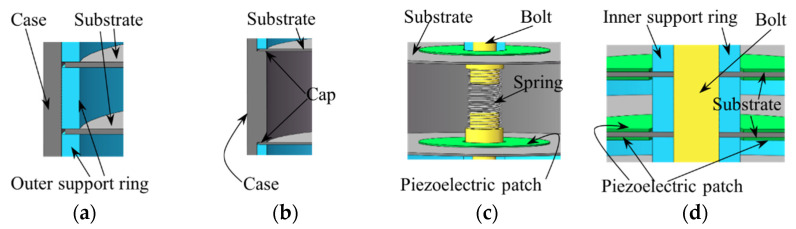
Local enlarged view of points *A*, *B*, *C*, and *D* in Figure 2. (**a**) Local view of point *A*; (**b**) local view of point *B*; (**c**) local view of point *C*; (**d**) local view of point *D*.

The structure design and working mechanism of the new type of piezoelectric damper are displayed in Figure 1, Figure 2 and Figure 3. The PCD is an aluminum alloy substrate disc bonded with one/two concentric circular piezoelectric patch of PZT4 (Lead Zirconate Titanate) by epoxy resin (shown in Figure 1a). The normal section of unimorph PCD is displayed in Figure 1b, in which a PZT4 patch with an inner diameter of *d_p_*, an outer diameter of *D_p_* and a thickness of *t_p_* is pasted on one surface of the concentric substrate disc. The normal section of bimorph PCD is revealed in Figure 1c, in which two PZT4 patches are pasted on the two surfaces of the concentric substrate disc with an inner diameter of *d_s_*, an outer diameter of *D_s_* and a thickness of *t_s_*. In this experiment, the array of PCDs shown as Figure 2a has the actions of a damper and spring, and can absorb and transfer part of the vibration energy from the outer excitation into electric energy to attenuate the outer vibration. It is obvious that two arrays of PCDs shown in Figure 2a need to match two arrays of inner supporting rings shown in Figure 2b, two arrays of outer supporting rings shown in Figure 2c, and a load transfer unit shown in Figure 2d to form a piezoelectric damper shown in Figure 2e. The inner and outer support rings are used to isolate the PCDs and fix them in an array, which can be seen in Figure 2e. The load transfer unit is composed of two pilot bearings, two nuts, a spring, and two bolts. The pilot bearings, inlaid in the concentric holes of the top and bottom covers, can make the bolts just move in the axial direction and reduce the friction between bolts and covers. Two pairs of nuts on the bolts are used to make the array of PCDs move with the bolts. The installation of these components is displayed in Figure 2e. The outer support rings are in contact with the inner surface of the cylinder case shown in Figure 3a. The PCDs are isolated by inner and outer support rings and restricted by the cap (Figure 3b), the covers and the nuts. The outer excitation is exerted on the array of PCDs through the load transfer unit. The axial displacement of the bolts is restricted in a range by the spring (Figure 3c) connecting the two bolts, which can protect the PCDs to avoid broken. The inner support ring shown in Figure 3d circles around the bolt and make PCDs has a uniform deformation in the move of the bolts.

## 3. Experimental Study

The dimensions and physical parameters of substrates S1 and S2 in the experiments are revealed in Table 1, including inner diameter, outer diameter, thickness, density, and damping ratio.

The inner diameter, outer diameter, thickness, Young’s modulus, shear modulus, passion’s ratio, and capacitance of PZT4 patches of P1–P8 are specifically listed in Table 2.

The combination style, experimental specimen, the weight, and the stiffness of PCD units from 1# to 11# are displayed in Table 3.

**Figure 4:**
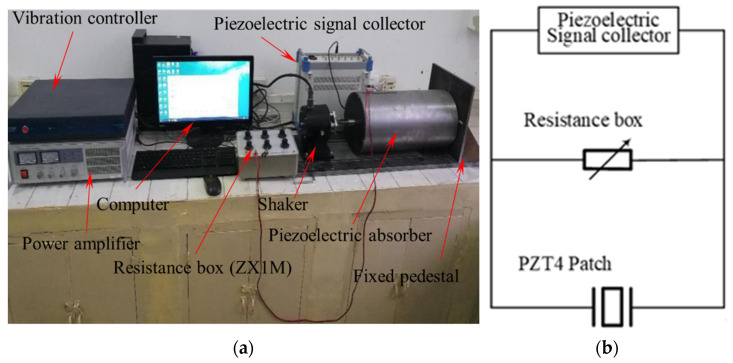
The experiment setup and electric circuit of the piezoelectric damper. (**a**) experiment setup; (**b**) electric circuit.

Specially, in order to study the influence of the outer diameter of the PZT4 patch on the energy harvesting ability of the PCD unit, PCDs of 1#–5# are fabricated by pasting PZT4 patches of P1–P5 on one surface of the substrate of S1, respectively. The PZT4 patches of P1–P5 have the same inner diameter of 20 mm, the thickness of 1 mm, and different outer diameters in turn of 60 mm, 65 mm, 70 mm, 75 mm, and 80 mm. Similarly, to study the effect of the inner diameter on the energy harvesting behavior of the piezoelectric unit, PCDs of 5#–8# are manufactured through pasting PZT4 patches of P5–P8 on one surface of the substrate S1, respectively. The PZT4 patches of P5–P8 have the same outer diameter of 80 mm, thickness of 1 mm, and different inner diameter in turn of 20 mm, 25 mm, 30 mm, and 35 mm. In order to research the effect of the substrate thickness on the energy harvesting behavior of the piezoelectric unit, a PZT4 patch with an inner/outer diameter of 35 mm/80 mm and a thickness of 1 mm is pasted on the surfaces of the substrates with a thickness of 0.6 mm and 1 mm to form the PCDs of 8# and 9#, respectively. To study energy harvesting behavior of the bimorph PCD, four PZT4 patches of P8 are pasted on two sides of the substrates of S1 and S2 to form PCD units of 10# and 11#, the output voltage and power from PCD units of 10# and 11# will be compared with those from PCD units of 8# and 9#, respectively. The outer surface of the PZT4 patch is connected with a wire as a positive electrode, and the inner surface between the PZT4 patch and the substrate is connected with a copper foil as a negative electrode, then the copper foil is connected with a wire. The experimental model of the piezoelectric damper is fixed on the fixed pedestal shown in Figure 4. In the experiment, the sine wave signal of the shaker (SA-JZ040) is generated by the vibration controller (VT-9008) and amplified by the power amplifier (SA-PA010). The piezoelectric signal collector (SC-HY-PZT-2.0, ±50 V, ±1%) is used to record the harvested electric signal, and the resistor box (ZX1M 0–1000 ΚΩ, ±0.1%) in the circuit aims for adjusting the load resistance of the access circuit to achieve the maximum output power. The outer vibration is transferred to the PCDs through the bolt to make the PZT4 patch strain and generate electricity in the vibration. Furthermore, the vibration controller (VT-9008) can control the acceleration, displacement and speed of the shaker at different frequencies. It is noted that in the following experiments the excitation acceleration is set to be 1.5 g and the excitation amplitude is set to be 5 mm.

## 4. Results and Analysis

### 4.1. The Effect of the Outer Diameter of the PZT4 Patch

**Figure 5:**
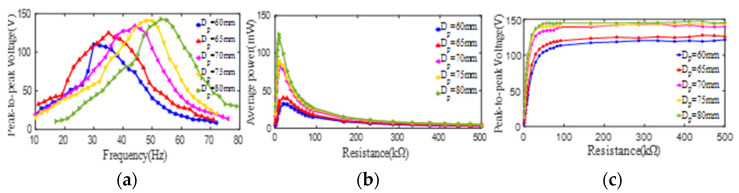
The effect of the outer diameter of the PZT4 patch on its peak-to-peak voltage and average power. (**a**) peak-to-peak voltage versus excitation frequency; (**b**) average power versus load resistance; (**c**) peak-to-peak voltage versus load resistance.

The peak-to-peak voltages (open-loop) of 1#–5# PCDs in different excitation frequencies are displayed in Figure 5a. It can be observed from Figure 5a that the peak-to-peak voltages and the 1st resonant frequencies of the unimorph PCDs all show a trend of increase with the increase of the outer diameter of the PZT4 patches. Specifically, when the outer diameter of the PZT4 patch is taken as 60 mm, 65 mm, 70 mm, 75 mm, and 80 mm, the maximum peak-to-peak voltage and the corresponding 1st resonant frequency of the PCD are (110 V, 30 Hz), (125 V, 35 Hz), (134 V, 44 Hz), (141 V, 48 Hz), and (143 V, 53 Hz), respectively. This phenomenon indicated that in the same excitation amplitude condition, the strain born by the PZT4 patch increases with the increase of its outer diameter, which leads to the increase of the voltage from the PZT4 patch. At the same time, with the increase of the outer diameter, the bending rigidity of the PCD increases, which causes the increase of the natural frequency of the PZT4 patch. In the 1st resonance, the changes of the average powers of 1#–5# PCDs along the increase of the load resistance are presented in Figure 5b. As displayed in Figure 5b, with the increase of the outer diameter of the PZT4 patch as 60 mm, 65 mm, 70 mm, 75 mm, and 80 mm, the maximum average powers from 1#–5# unimorph PCDs are 32 mW, 40 mW, 78 mW, 89 mW, and 125 mW corresponding to the optimal load resistances of 19.61 KΩ, 19.61 KΩ, 9.9 KΩ, 9.9 KΩ, and 8.9 KΩ, respectively. Obviously, the average output powers from 1#–5# PCDs all sharply increase up to their respective peaks and then sharply decrease till the load resistance increases to 100 KΩ, and then the increase of the load resistance hardly affects the average output powers from 1#–5# PCDs. In addition, in the 1st resonance, the peak-to-peak voltages (close-loop) of 1#–5# PCDs are measured in the different load resistance and the corresponding results are illustrated in Figure 5c, from which it can be observed that the voltages from 1#–5# PCDs all increase sharply with the increase of the load resistance (resistance box) from 0.9 KΩ to 19 KΩ, and then tend to a stable value with the increase of the load resistance from 19 KΩ to 500 KΩ. This phenomenon is mainly because with the increase of the load resistance from 0.9 KΩ to 19 KΩ, the proportion of the load resistance in the total resistance of the circuit rapidly increases, and after 19 KΩ, the inner resistance of the PZT4 patch is insignificant compared to the load resistance. Namely, inner resistance of the PZT4 patch has a negligible effect on the circuit current and the voltage exerting on the load resistance after the load resistance is greater than 19 KΩ. This process can also be explained by Equation (1) given below. Based on the discussion above, a conclusion can be found that the peak-to-peak voltage (open/close-loop) and the maximum average power, and the 1st natural frequency of the PCD all increase with the increase of the outer diameter of the PZT4 patch from 60 mm to 80 mm.

(1) $$V_{L}=IR_{L}=\frac{R_{L}}{R_{I}+R_{L}}V,$$

In which, *V* and *I* denote the total voltage and the current in the electric circuit shown as Figure 4b; *R_L_* and *V_L_* express the load resistance and its voltage; *R_I_* is the inner resistance of the PZT4 patch.

### 4.2. The Effect of the Inner Diameter of the PZT4 Patch

**Figure 6:**
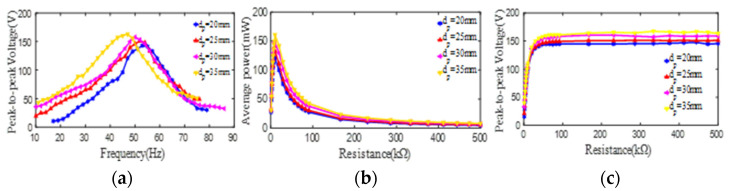
The effect of the inner diameter of the PZT4 patch on its peak-to-peak voltage and average power. (**a**) peak-to-peak voltage versus excitation frequency; (**b**) average power versus load resistance; (**c**) peak-to-peak voltage versus load resistance.

Following the research on the effect of the outer diameter of the PZT4 patch, the effect of its inner diameter on its output electric parameters is studied. The peak-to-peak voltages (open-loop) from unimorph PCDs of 5#–8# at different excitation frequencies are measured and displayed in Figure 6a, in which the trend of the curves of 5#–8# PCDs are roughly the same, increasing firstly up to the peak and then decrease continually. As Figure 6a, the maximum peak-to-peak voltages and their corresponding 1st resonant frequencies are (143 V, 53 Hz), (151 V, 52 Hz), (158 V, 50 Hz), and (163 V, 47 Hz) corresponding to the inner diameter of 20 mm, 25 mm, 30 mm, and 35 mm, respectively. These data indicate that with the increase of the inner diameter of the PZT4 patch, the maximum voltage can be improved to some extent. This is because with the increase of the inner diameter (from 20 mm to 35 mm), the bending rigidity of the PCD decreases, which leads to the increase of the strain and output voltage of the PZT4 patch indicated as Equations (2)–(4). The average powers of 5#–8# PCDs all show a sharply linear increase up to the peak value along the increase of the load resistance and then a sharply nonlinear decrease followed by a gentle down, which can be seen in Figure 6b. Meanwhile, it can be found the maximum average power of the unimorph PCD increases with the increase of the inner diameter of the PZT4 patch. Specifically, for the inner diameter of 20 mm, 25 mm, 30 mm, and 35 mm, the maximum average powers and the corresponding optimal load resistances are (125 mW, 8.9 KΩ), (141 mW, 8.9 KΩ), (148 mW, 9.9 KΩ), and (160 mW, 9.9 KΩ), respectively. According to Figure 5c, it is obvious that the peak-to-peak voltages (close-loop) from unimorph PCDs of 5#–8# also show a sharply linear increase up to the maximum and then basically keep it with the increase of the load resistance. In addition, it can be observed that the output voltages of the unimorph PCDs increase with the increase of the inner diameter of the PZT4 patch when the load resistance changes from 9.9 KΩ to 500 KΩ.

In a word, from Figure 6, it can be observed that with the increase of the inner diameter of the PCD, its peak-to-peak voltage (open/close-loop) and maximum average power increase, but the 1st natural frequency of the PCD decreases.

(2) $$V(t)=Q(t)/C=d_{31}\varepsilon (t)E_{p}t_{p}/\varepsilon_{0}\varepsilon_{r},$$

(3) $$Q(t)=d_{31}\varepsilon (t)E_{p}A_{p},$$

(4) $$C=\varepsilon_{0}\varepsilon_{r}A_{p}/t_{p},$$

where, *Q*(*t*), *V*(*t*) and *ε*(*t*) are the generated electric charge, voltage for an open-loop circuit, and strain at time *t* from the PZT4 patch; *C* is the capacitance of the PZT4 patch; *d*_31_ is the piezoelectric constant; *E_p_*, *A_p_*, and *t_p_* are the Young’s modulus, surface area, and thickness of the PZT4 patch; *ε_0_* is the permittivity of air and *ε_r_* is the dielectric constant of the PZT4 patch.

### 4.3. The Effect of the Thickness of the Substrate

**Figure 7:**
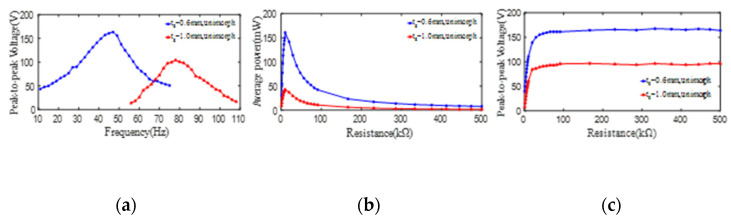
The effect of the substrate thickness on the peak-to-peak voltage and average power of one PZT4 patch bonded on the substrate. (**a**) peak-to-peak voltage versus excitation frequency; (**b**) average power versus load resistance; (**c**) peak-to-peak voltage versus load resistance.

In order to study the influence of the substrate thickness on the output electric parameters of unimorph PCD, the same PZT4 patches with an inner diameter of 35 mm, an outer diameter of 80 mm, and a thickness of 1 mm are bonded on the substrates with a thickness of 0.6 mm and 1.0 mm to form PCDs of 8# and 9#, respectively. Shown as Figure 7a, the maximum peak-to-peak voltages (open-loop) from unimorph PCDs of 8# and 9# can reach up to 163 V and 104 V when they work at the 1st resonances of 47 Hz and 78 Hz, respectively. To further investigate the influence of the load resistance on the average output power and the peak-to-peak voltage from PCDs, the parallel resistance with a measuring range of 0.9 kΩ to 500 kΩ is introduced to the experimental circuit. The maximum average powers from unimorph PCDs of 8# and 9# all sharply increase up to their respective peaks and then sharply decrease along the increase of the load resistance till 100 KΩ, and then show a gently reducing tendency, which is displayed in Figure 7b. Furthermore, it can be obtained that the maximum average power from unimorph PCDs of 8# and 9# and the corresponding optimal load resistance are (161 mW, 9.9 kΩ) and (42 mW, 9.9 kΩ), respectively. Shown as Figure 7c, the peak-to-peak voltages (close-loop) from unimorph PCDs of 8# and 9# sharply increase up to 159 V and 98 V firstly and then basically keep a constant with the increase of the load resistance.

In short, with the increase of the substrate thickness from 0.6 mm to 1.0 mm, the peak-to-peak voltages (close-loop) from unimorph PCDs and the average power decrease. However, the 1st natural frequency of the PCD increases.

**Figure 8:**
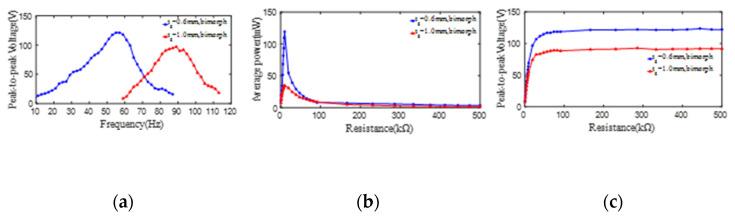
The effect of the substrate thickness on the peak-to-peak voltage and average power of two PZT4 patches bonded on the substrate. (**a**) peak-to-peak voltage versus excitation frequency; (**b**) average power versus load resistance; (**c**) peak-to-peak voltage versus load resistance.

To further study the effect of the substrate thickness on the output peak-to-peak voltage and average power from PZT4 patch of bimorph PCD, the experimental results of the voltages and powers from the bimorph PCD with a substrate thickness of 0.6 mm (10#) and 1 mm (11#) are taken out for analysis. The changing of the peak-to-peak voltages (open-loop) from one PZT4 patch along the increase of the excitation frequency is revealed in Figure 8a, in which the relationship curves all similar to a hill with the maximum peak-to-peak voltage of 121 V at 55 Hz for substrate thickness of 0.6 mm and 97 V at 89 Hz for substrate thickness of 1 mm. This phenomenon once again indicates that the PCD with the thinner substrate is better for generating a larger peak-to-peak voltage. In order to further investigate the influence of load resistance on the output power and peak-to-peak voltage from bimorph PCD of the piezoelectric damper. The maximum output powers of the PCDs are extracted and shown in Figure 8b, in which the powers all increase firstly and then decrease with the increase of the load resistance. Furthermore, it can be found that the output power and corresponding optimal resistance from the bimorph PCDs of 10# and 11# are (119 mW, 9.9 kΩ) and (34 mW, 9.9 kΩ), respectively. The peak-to-peak voltages (close-loop) from the bimorph PCDs of 10# and 11# in different load resistance are measured and displayed in Figure 8c, in which the curves show an increase sharply and then basically keep a stable value of 115 V for a substrate thickness of 0.6 mm and 87 V for substrate thickness of 1 mm.

Obviously, for bimorph PCD, its peak-to-peak voltages (open/close-loop), average power, and 1st natural frequency have the same changing rule as unimorph PCD with the increase of the substrate thickness.

### 4.4. The Effect of the Unimorph/Bimorph PCD

**Figure 9:**
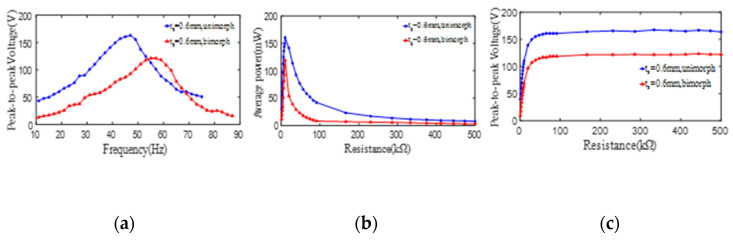
The effect of unimorph PCD and bimorph PCD on the peak-to-peak voltage and average power of one PZT4 patch (*t_s_* = 0.6 mm). (**a**) peak-to-peak voltage versus excitation frequency; (**b**) average power versus load resistance; (**c**) peak-to-peak voltage versus load resistance.

In order to investigate the effect of the unimorph PCD and bimorph PCD on their energy harvesting ability, the peak-to-peak voltages and average powers from the unimorph and bimorph PCDs (*d_p_* = 35 mm, *D_p_* = 80 mm, *t_s_* = 0.6 mm) are extracted and revealed in Figure 9. Figure 9a demonstrated that the voltage (open-loop) changes similar to a gentle hill along the increase of the excitation frequency from 10 Hz to 90 Hz. Obviously, the voltage from unimorph PCD is larger than the corresponding one from bimorph PCD; meanwhile, the 1st resonant frequency of the unimorph PCD is less than that of the bimorph PCD. The maximum peak-to-peak voltages (open-loop) from one PZT4 patch of unimorph and bimorph PCDs are 163 V and 121 V at the 1st resonant frequencies of 47 Hz and 55 Hz, respectively. It is noted that the difference between the peak-to-peak voltages from unimorph PCD and bimorph PCD can reach up to 42 V. This phenomenon is because the unimorph PCD has a smaller bending rigidity and a bigger deformation than that of bimorph PCD. Figure 9b displayed the average powers of the unimorph and bimorph PCDs increase similar to a steep mountain along the increase of the load resistance from 0.9 kΩ to 100 kΩ and then attenuate close to zero value. It can be found that the average output powers from one PZT4 patch of unimorph and bimorph PCDs can reach up to 161 mW and 119 mW at the same optimal resistance of 9.9 kΩ. Obviously, the difference of the maximum average powers is up to 41 mW. Of course, the total average output powers of the two PZT4 patches (whether in series or parallel) of bimorph PCD is far bigger than that of unimorph PCD, but it is at the expense of inputting bigger excitation force. As shown in Figure 9c, with the increase of the load resistance, the peak-to-peak voltages (close-loop) from one PZT4 patch show a steep rising up to the peak of 159 V and 115 V for unimorph and bimorph PCDs and then tend to a steady value.

**Figure 10:**
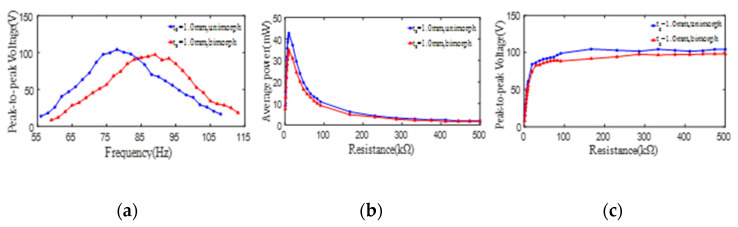
The effect of unimorph PCD and bimorph PCD on the output peak-to-peak voltage and average power of one PZT4 patch (*t_s_* = 1 mm). (**a**) peak-to-peak voltage versus excitation frequency; (**b**) average power versus load resistance; (**c**) peak-to-peak voltage versus load resistance.

To further explore the influence of unimorph and bimorph PCDs, the peak-to-peak voltages and average powers from unimorph PCD (9#) and bimorph PCD (11#) with a substrate thickness of 1 mm are also extracted and revealed in Figure 10. It can be obtained from Figure 10a that the peak-to-peak voltages (open-loop) from the unimorph and bimorph PCDs (*d_p_* = 35 mm, *D_p_* = 80 mm, *t_s_* = 1 mm) can reach up to 104 V at its 1st resonant frequency of 78 Hz and 97 V at its 1st resonant frequency of 89 Hz. This phenomenon is also caused by the smaller bending rigidity of the unimorph PCD compared to that of bimorph PCD. In addition, the maximum average power from unimorph and bimorph PCDs can reach up to 42 mW and 34 mW, respectively, at the same optimal resistance of 9.9 kΩ, which can be seen in Figure 10b. It also can be found from Figure 10c that the peak-to-peak voltages (close-loop) from unimorph PCD (9#) and bimorph PCD (11#) sharply rise up to a stable value of 104 V for unimorph PCD and 97 V for bimorph PCD with the increase of the load resistance and then basically keep a constant. From Figure 10, it can be found that the maximum peak-to-peak voltages or the maximum average powers from the unimorph PCD are slightly larger than the corresponding ones from the bimorph PCD. Specially, the difference of the maximum peak-to-peak voltages is 7 V, and the difference between the maximum average powers is 8 mW, which is far less than the corresponding difference between the voltages and the average powers from PCDs with a substrate thickness of 0.6 mm. This phenomenon indicates that with the increase of the substrate thickness, the energy harvesting ability of one PZT4 patch from bimorph PCD is gradually close to that from unimorph PCD. That is to say, with the increase of the substrate thickness, the energy harvesting ability of bimorph PCD is basically two times that of unimorph PCD. At the same time, by comparing with the experimental results in the last paragraph, it is obvious that when the substrate thickness is 0.6 mm, unimorph/bimorph PCD has a bigger difference in energy harvesting shown in Figure 9c and Figure 10c.

## 5. COMSOL Simulation

**Figure 11:**
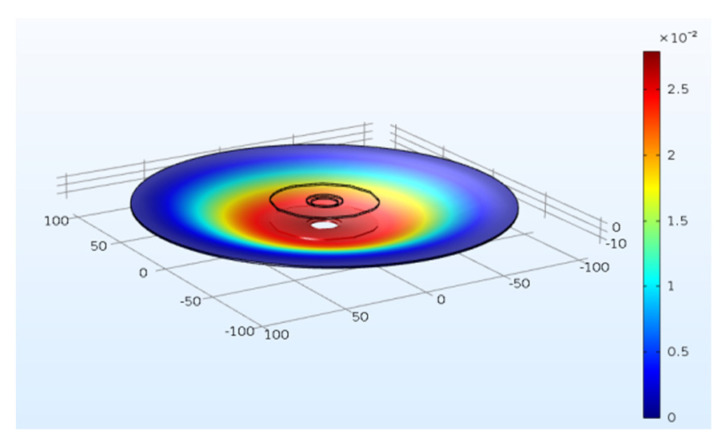
The stress contour of the PCDs.

**Figure 12:**
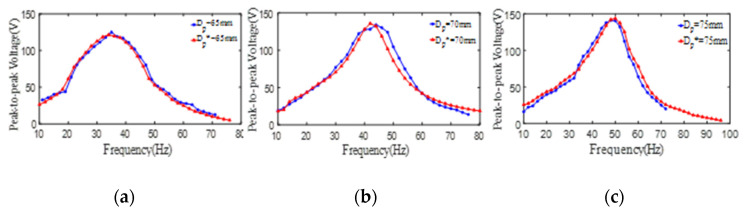
The relationship curves of the peak-to-peak voltage and the excitation frequency of the unimorph PCD from experimental results (blue line) and simulation results (red line). (**a**) peak-to-peak voltage of PCD with *D_p_* = 65 mm; (**b**) peak-to-peak voltage of PCD with *D_p_* = 70 mm; (**c**) peak-to-peak voltage of PCD with *D_p_* = 75 mm.

In order to comprehensively research the effects of the inner/outer diameter and the thickness of the PZT4 patch and the substrate thickness on the energy harvesting behavior of the PCDs, more PCD models with different dimension are built by COMSOL software. To visually display the stress distribution of the PCD under the axial force, the stress contours of the PCD are simulated by COMSOL software and revealed in Figure 11. It can be intuitively found that the deformation focuses on the area bonding PZT4 patch and its neighborhood. In addition, to verify the accuracy and the reliability of the COMSOL models, the peak-to-peak voltages of the experimental models of 2#–4# PCDs are simulated by corresponding COMSOL models. The results from experiments and simulations are extracted and shown in Figure 12. It can be found from Figure 12 that the curves of the peak-to-peak voltages from the simulation are roughly the same as those from the experiment, which can be seen in Figure 12a–c for 2#, 3#, and 4# PCD, respectively. These phenomena indicate that the simulation models of PCDs are reliable and the simulation results of the voltages of PCDs are accurate. Therefore, using COMSOL software to extend the research of PCDs with more dimensions is feasible. Just to be clear, in the following description, the size parameters of PCDs with star mean the simulation models. For example, D_p_^*^ denotes the outer diameter of the PZT4 patch for COMSOL simulation models.

**Figure 13:**
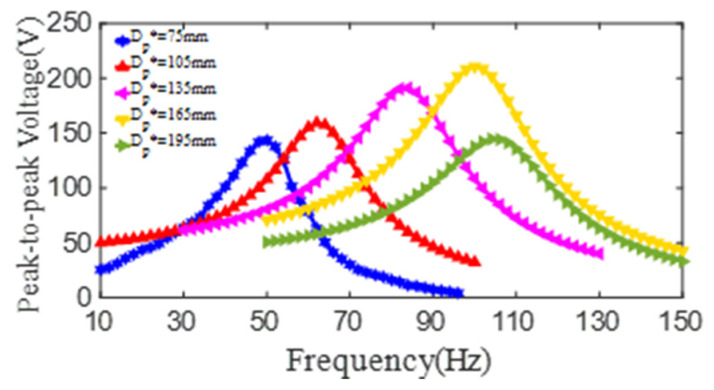
The relationship curves of the peak-to-peak voltage and the excitation frequency of PZT4 patch with different outer diameter (d_p_^*^ = 20 mm, t_p_^*^ = 1 mm, t_s_^*^ = 0.6 mm).

In order to further explore the effect of the outer diameter of the PZT4 patch on the output voltage of the PCD, a series of PCD models are built by COMSOL software. In these models, the material properties are taken as those of the experimental specimens shown in Table 1 and Table 2, the dimensions of the PCDs are set to be: d_p_^*^ = 20 mm, t_p_^*^ = 1 mm, t_s_^*^ = 0.6 mm, d_s_^*^ = 15 mm, and D_s_^*^ = 218 mm, in addition, the outer diameters of the PZT4 patch include 75 mm, 105 mm, 135 mm, 165 mm, and 195 mm. According to the simulation, the output voltages of each PCD model all show an increase first and then decrease with the increase of the excitation frequency, which can be seen in Figure 13. The maximum peak-to-peak voltage and the corresponding 1st resonant frequency are (143 V, 50 Hz), (159 V, 62 Hz), (191 V, 84 Hz), (210 V, 100 Hz), and (144 V, 104 Hz) for the PZT4 patch with an outer diameter of 75 mm, 105 mm, 135 mm, 165 mm, and 195 mm, respectively. Obviously, the maximum peak-to-peak voltages increase with the increase of the outer diameter from 75 mm to 165 mm and then decrease when the outer diameter is more than 165 mm. Meanwhile, it can be found that the 1st resonant frequency of the PCD keeps a continual increase with the increase of the outer diameter of the PZT4 patch. This phenomenon indicates that there is an optimal outer diameter of the PZT4 patch for the PCD to output maximum voltage, rather than the bigger diameter of the PZT4 patch is always better for the PCD to output maximum voltage.

Based on the test and simulation results, the formula for the peak-to-peak voltages (*V_pp_*) of the PCD and the outer diameter of *D_p_* can be fitted as below:

(5) $$V_{pp}=552-12D_{p}+0.11D_{p}^{2}+3.12*10^{-4}D_{p}^{3}(D_{p}=75\sim 195\mathrm{mm})$$

**Figure 14:**
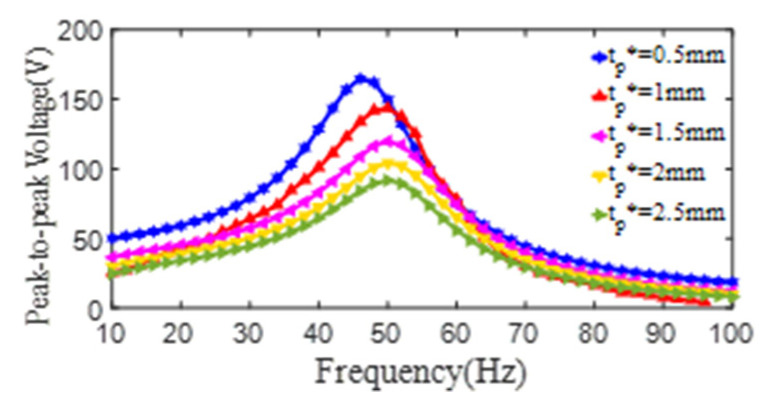
The relationship curves of the peak-to-peak voltage and the excitation frequency of PZT4 patch with different thickness (d_p_^*^ = 20 mm, D_p_^*^ = 75 mm, t_s_^*^ = 0.6 mm).

To explore the effect of the thickness of the PZT4 patch on the energy harvesting behavior of the PCD, the simulation models of the damper, in which the PZT4 patch with different thicknesses of t_p_^*^, are built. In these simulations, the material properties are displayed in Table 1 and Table 2, and the dimensions are taken as d_p_^*^ = 20 mm, D_p_^*^ = 75 mm, t_s_^*^ = 0.6 mm, d_s_^*^ = 15 mm, and D_s_^*^ = 218 mm. As shown in Figure 14, corresponding to the thickness t_p_^*^ of 0.5 mm, 1.0 mm, 1.5 mm, 2.0 mm, and 2.5 mm, the maximum peak-to-peak voltages and the corresponding 1st resonant frequency are, respectively (164 V, 46 Hz), (143 V, 49 Hz), (119 V, 50 Hz), (104 V, 50 Hz), and (91 V, 51 Hz). The curves of the peak-to-peak voltage and the excitation frequency all increase firstly to a maximum value and then decrease. It can be concluded that the peak-to-peak voltages decrease with the increase of the thickness of the PZT4 patch, the reason is that with the increase of the thickness of the PZT4 patch, the stiffness of the PCD increase, which leads to the notable decrease of the strain and output voltage of the PZT4 patch although its thickness increases (indicated by Equation (1)).

For the convenience of evaluating the effect of the PZT4 patch thickness on the output voltage of the PCD, the peak-to-peak voltage (*V_pp_*) of the PCD and the corresponding thickness of *t_p_* are extracted and fitted as the formula as follows:

(6) $$V_{pp}=192.2-58.43t_{p}+7.14t_{p}^{2}(t_{p}=0.5\sim 2.5\mathrm{mm}),$$

**Figure 15:**
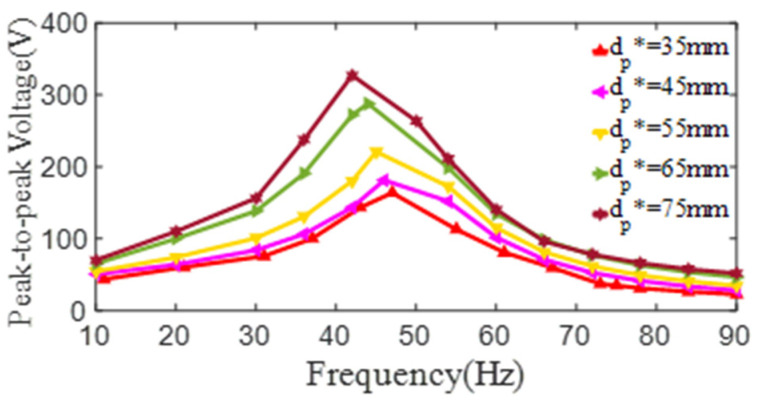
The relationship curves of the peak-to-peak voltage and the excitation frequency of PZT4 patch with different inner diameter (D_p_^*^ = 80 mm, t_p_^*^ = 1 mm, t_s_^*^ = 0.6 mm).

To expand the study on the relationship of the inner diameter of the PZT4 patch and the output voltage of the PCD, a series of simulation models of the damper are modeled, in which the substrate discs are bonded by PZT4 patches with different inner diameters. In these simulations, the dimensions of PZT4 patch and substrate are set to be D_p_^*^ = 80 mm, t_p_^*^ = 1 mm, t_s_^*^ = 0.6 mm, d_s_^*^ = 15 mm, and D_s_^*^ = 218 mm. It can be obtained from Figure 15 that the maximum peak-to-peak voltages and the 1st resonant frequency are (163 V, 47 Hz), (181 V, 46 Hz), (221 V, 45 Hz), (288 V, 44 Hz), and (327 V, 42 Hz), when the inner diameter d_p_^*^ of the PZT4 patch increases as 35 mm, 45 mm, 55 mm, 65 mm, and 75 mm, respectively. Obviously, with the increase of the inner diameter, the peak-to-peak voltage increases. This phenomenon is caused by the decrease of the bending rigidity of the PCD, which further causes the increases of the surface strain and the output voltage of the PZT4 patch. Another finding is that with the increase of the inner diameter, the 1st resonant frequency of the PCD keeps a continuous decrease. This is the same as the rule in experiments and for the same reason as experiments.

To theoretically value the effect of the inner diameter of the PZT4 patch on the output electric parameter of the PCD, the peak-to-peak voltage of the PCD corresponding to the inner diameter of *d_p_* is extracted and their relationship is fitted as the formula as below:

(7) $$V_{pp}=614-9.77d_{p}+0.049d_{p}^{2}(d_{p}=35\sim 75\mathrm{mm}),$$

**Figure 16:**
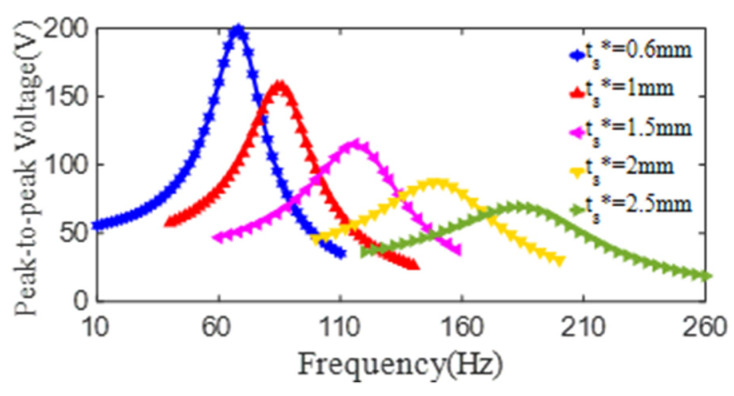
The relationship curves of the peak-to-peak voltage and the excitation frequency of PZT4 patch with different substrate thickness (d_p_^*^ = 80 mm, D_p_^*^ = 135 mm, t_p_^*^ = 1 mm).

The effect of the substrate thickness on the output voltage of the PCD is also further researched by the simulation method. In this computation process, the parameters are taken as d_p_^*^ = 80 mm, D_p_^*^ = 135 mm, t_p_^*^ = 1 mm, d_s_^*^ = 15 mm, and D_s_^*^ = 218 mm; and the substrate thickness is changed from 0.6 mm to 2.5 mm. As displayed in Figure 16, when the substrate thickness of t_s_^*^ is rising to 0.6 mm, 1.0 mm, 1.5 mm, 2.0 mm and 2.5 mm, the maximum peak-to-peak voltage from the PZT4 patch is 201 V at 65 Hz, 163 V at 82 Hz, 121 V at 115 Hz, 91 V at 150 Hz and 71 V at 180 Hz, respectively. From the data above, it can be founded that as the thickness of the substrate increases, the 1st resonant frequency of the PCD increases markedly, but the peak-to-peak voltage at this frequency decreases. Obviously, with the increase of its substrate thickness, the bending stiffness and the natural frequency of the PCD increases. Another key reason is that the excitation amplitude of the shaker decreases with the increase of its excitation frequency, which also leads to the decreases of the deformation and the generated voltage (at 1st resonance) of the PZT4 patch of the PCD with a bigger substrate thickness.

On the basis of the experiment and simulation, the relationship curve of the peak-to-peak voltage of the PZT4 patch of the PCD and the substrate thickness of *t_s_* can be fitted using the equation as below:

(8) $$V=253-107.54t_{s}+13.71t_{s}^{2}(t_{s}=0.6\sim 2.5\mathrm{mm}),$$

## 6. Discussion

The piezoelectric damper proposed in this research is practical and ingenious. Firstly, the proposed damper can ingeniously attenuate the vibration by transferring part of the vibration energy into electric power for recycling utilization; Secondly, the restrained condition of the PCD and the force style make its strain uniform and focus on the piezoelectric coupling area shown as Figure 11; Thirdly, the design of the array PCDs make the damper have a great potential to attenuate the vibration and generate electric power. The key highlight for this damper is that it can bear a huge impact and output considerable electric energy for the huge bending rigidity of the array PCDs. The bearing capacity and electric generation capacity of the damper can be adjusted to suit any practical requirement by changing the number and the dimension of the PCD in the array. In addition, it is worth mentioning that the ingenious design of the damper makes the small PZT4 patch play a great role, which makes the piezoelectric damper can be used in many fields, including civil structure or mechanical structure, and generates appreciable electric energy. For example, the total output power of the piezoelectric damper including 20 of unimorph PCDs can reach up to 3.22 W, in which the average output power and power density of one PZT4 patch (*D_p_* = 80 mm, *d_p_* = 35 mm, and *t_p_* = 1 mm) can reach up to 161 mW and 39.61 μW/mm^3^. This density is larger than the maximum output power densities from L-shaped harvester (2.1 μW/mm^3^) [33] and U-shaped harvester (28.1 μW/mm^3^) [34]. Of course, with the increase of the number and/or the dimension of the PCD, the bearing capacity and electric generation capacity of the damper can be improved further to meet the vibration attenuation of large-scale structures.

However, the PCD of the proposed damper just can bear a small axial displacement, this restraint makes the damper just to be used in the small deformation or displacement situation. For example, the axial movement of the damper in this design should be on the millimeter scale. Otherwise, the PCD will be broken by the overrun strain/stress. In view of this, in our next research, a new experiment will be conducted by replacing the outer/inner supporting ring with an outer/inner supporting spring with the same stiffness to increase the axial movement range of the PCD in the damper. Meanwhile, the damping performance of the damper will also be studied.

## 7. Conclusions

A new type of piezoelectric damper, including two PCD arrays, two inner support ring arrays, two outer support ring arrays, and a load transfer unit, is developed. A series of forced-vibration experiments about the piezoelectric damper are conducted and comprehensively simulation research is carried out, in which the effects of the design dimensions of the damper, unimorph/bimorph PCD, and the load resistance on the peak-to-peak voltage and average power are acquired and analyzed. According to the research results, some important conclusions can be drawn as follows.

With the increase of the outer diameter of the PZT4 patch from 60 mm to 80 mm, the peak-to-peak voltage and average power from the unimorph PCD always increase, when the outer diameter of the piezoelectric patch increase from 80 mm to 195 mm, the peak-to-peak voltage increase up to the peak firstly and then decrease.

With the increase of the inner diameter of the PZT4 patch from 20 mm to 75 mm, the peak-to-peak voltage and average power from the unimorph PCD always increase.

In the condition of the same excitation and design parameters, the unimorph PCD is better than bimorph for generating larger peak-to-peak voltage and average output power.

In the condition of the same excitation, the PCD with a thinner substrate thickness is better to generate a bigger peak-to-peak voltage and average output power.

With the increase of the substrate thickness, the difference of the peak-to-peak voltages and the average powers from the PZT4 patch of unimorph PCD and bimorph PCD decrease.

A peak-to-peak voltage of 163 V and an average power of 161 mW can be achieved from a PZT4 patch of a unimorph PCD (*D_p_* = 80 mm, *d_p_* = 35 mm, and *t_p_* = 1 mm), a total output power of 3.22 W can be obtained for a proposed damper including 20 of unimorph PCDs.

## Figures and Tables

**Table 1:** Dimensions and Physical parameters of substrates.

Substrates	Inner Diameter *d_s_* (mm)	Outer Diameter *D_s_* (mm)	Thickness *t_s_* (mm)	Density (kg/m^3^)	Damping Ratio ξ
S1	15	218	0.6	2650	0.0319
S2	15	218	1	2650	0.0478

**Table 2:** Dimensions and physical parameters of PZT4 patches.

PZT4 Patch	Inner Diameter *d_p_* (mm)	Outer Diameter *D_p_* (mm)	Thickness *t_p_* (mm)	Young’s Modulus E (Gpa)	Shear Modulus E_s_ (Gpa)	Poisson’s Ratio μ	Capacitance C (nF)
P1	20	60	1	69.49	22.61	0.337	38.14
P2	20	65	1	69.49	22.61	0.337	42.42
P3	20	70	1	69.49	22.61	0.337	50.2
P4	20	75	1	69.49	22.61	0.337	55.2
P5	20	80	1	69.49	22.61	0.337	63.3
P6	25	80	1	69.49	22.61	0.337	62.7
P7	30	80	1	69.49	22.61	0.337	62.2
P8	35	80	1	69.49	22.61	0.337	58.4

**Table 3:** Material properties of piezoelectric harvester units.

Piezoelectric Harvester Unit	Combination Styles	Experimental Specimen	Weight (g)	Stiffness(N/m)
1# (P1 + S1)	Unimorph	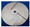	80.8	13,823
2# (P2 + S1)	Unimorph	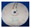	83.8	14,715
3# (P3 + S1)	Unimorph	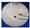	88.5	19,834
4# (P4 + S1)	Unimorph	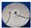	91.9	23,532
5# (P5 + S1)	Unimorph	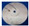	97.9	24,010
6# (P6 + S1)	Unimorph	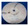	97.1	19,007
7# (P7 + S1)	Unimorph	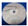	96.1	16,292
8# (P8 + S1)	Unimorph	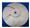	94.9	13,417
9# (P8 + S2)	Unimorph	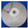	131.3	19,834
10# (P8 + S1)	Bimorph	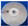	125.2	23,252
11# (P8 + S2)	Bimorph	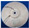	161.6	36,987
